# Electron Holes in G-Quadruplexes: The Role of Adenine Ending Groups

**DOI:** 10.3390/ijms222413436

**Published:** 2021-12-14

**Authors:** Evangelos Balanikas, Lara Martinez-Fernandez, Gérard Baldacchino, Dimitra Markovitsi

**Affiliations:** 1 LIDYL, CEA, CNRS, Université Paris-Saclay, 91191 Gif-sur-Yvette, France; vangelis.balanikas@cea.fr (E.B.); gerard.baldacchino@cea.fr (G.B.); 2 Departamento de Química, Modúlo 13, Facultad de Ciencias and IADCHEM (Institute for Advanced Research in Chemistry), Campus de Excelencia UAM-CSIC, Universidad Autónoma de Madrid, Cantoblanco, 28049 Madrid, Spain

**Keywords:** G-Quadruplexes, electron holes, photoionization, guanine radicals, oxidative damage, time-resolved spectroscopy, quantum chemistry

## Abstract

The study deals with four-stranded DNA structures (G-Quadruplexes), known to undergo ionization upon direct absorption of low-energy UV photons. Combining quantum chemistry calculations and time-resolved absorption spectroscopy with 266 nm excitation, it focuses on the electron holes generated in tetramolecular systems with adenine groups at the ends. Our computations show that the electron hole is placed in a single guanine site, whose location depends on the position of the adenines at the 3′ or 5′ ends. This position also affects significantly the electronic absorption spectrum of (G^+^)^●^ radical cations. Their decay is highly anisotropic, composed of a fast process (<2 µs), followed by a slower one occurring in ~20 µs. On the one hand, they undergo deprotonation to (G-H2)^●^ radicals and, on the other, they give rise to a reaction product absorbing in the 300–500 nm spectral domain.

## 1. Introduction

**Figure 1:**
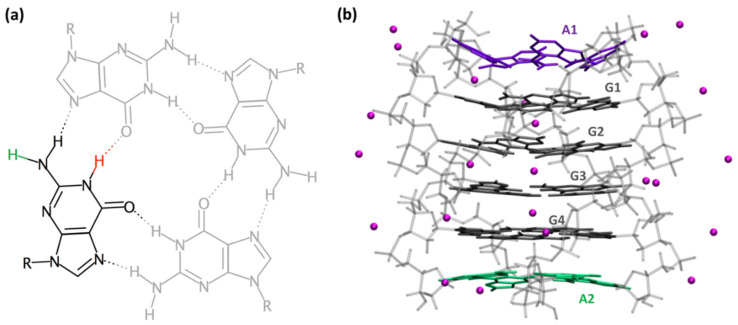
Schematic representation of a G-tetrad (**a**) and the (AG_4_A)_4_/K^+^ G-Quadruplex (**b**). Transfer of the red or green proton (**a**) to the aqueous solvent, concerning the guanine radical cation, gives rise to deprotonated radicals (G-H1)^●^ or (G-H2)^●^, respectively. In (**b**), guanines are depicted in gray (tetrads labelled as G1, G2, G3, G4), adenines in violet (at 5′, A1) and green (at 3′, A2); the backbone is shown in light grey and ions are represented by pink spheres.

Electron holes in DNA bases (radical cations) are generated through electron abstraction reactions with external oxidants or under the effect of ionizing radiation. Regardless of their initial location, electron holes are eventually trapped by guanine sites following a charge migration process [1,2,3,4,5,6,7,8,9]. Guanine sites constitute hot spots for oxidative damage because their oxidation potential is lower compared to that of other nucleobases [10], and it is further decreased upon stacking [4,5,6]. It is thus understandable that G-Quadruplexes, which are four-stranded structures characterized by vertical stacking of guanine tetrads (G-tetrads, Figure 1), are prone to such type of damage. Oxidative lesions may perturb the multiple biological functions in which these non-canonical structures play a key role, such as replication, transcription or genome maintenance [11]. Furthermore, G-Quadruplexes are targets for anticancer therapy, which may involve redox reactions [12]. Finally, G-Quadruplexes are extensively studied in view of applications in the field of nanotechnology [13], exploiting, in particular, their behavior as nanowires based on hole transport [14].

In the above-described context, the characterization of electron holes in G-Quadruplexes, (G^+^)^●^, is particularly important. On the one hand, it is expected to shed light on the primary steps leading to oxidative damage. On the other, it may contribute to the optimization of nanodevices based on charge transport. The first article reporting trapping of electron holes by G-Quadruplexes appeared in 2003 [15]. Later, femtosecond transient absorption experiments studied the efficiency of hole transport through such structures [16,17], while direct conductivity measurements were performed by integrating them in electric circuits [14,18,19]. In parallel, theoretical studies examined the role the G-Quadruplex topology on the conductivity and computed the electronic coupling which is the driving force for charge transport [14,20,21,22,23,24,25].

A different type of studies focused on the electron holes per se. Their absorption spectra were determined combining quantum chemistry calculations and nanosecond transient absorption spectroscopy [26,27]. Such experiments became possible thanks to the development of specific protocols allowing to study photo-activated processes in room temperature aqueous solutions of DNA multimers. Using low-intensity laser pulses (≤6 × 10^6^ W cm^−2^) at 266 nm, neither the backbone nor the solvent are photo-reactive and, consequently, the transient absorption (ΔA) signals recorded on the microsecond and millisecond time-scales stem, mainly, from nucleobase radicals [28,29,30] and/or photodimers [31,32]. However, an additional condition is indispensable in order to characterize spectrally the electron holes in G-Quadruplexes: the pH of the solution has to be adjusted to 3 to avoid deprotonation. As a matter of fact, (G^+^)^●^ are unstable in neutral aqueous solution losing a proton to the bulk water [33] (Figure 1a). However, measurements at pH 7 brought precious information regarding the deprotonation dynamics [34,35].

Among the G-Quadruplexes studied by nanosecond transient absorption, (AG_4_A)_4_/K^+^, formed by association of four AGGGGA strands in the presence of K^+^ ions, has the largest propensity to undergo low-energy photoionization [36]; the term “low-energy” is used in comparison with the ionization potential of the DNA constituents [37]. The quantum yield ϕ determined for one-photon ionization at 266 nm is 1.5 × 10^−2^, which is one order of magnitude higher than that of genomic DNA [29]. Interestingly, the location of the adenine groups at the 5′ or the 3′ ends was found to play a role in the photoionization process [36]. Although G-Quadruplexes occurring in vivo are formed by folding of a single strand, the tetramolecular systems presented here, in association with other analogs studied previously, constitute convenient models, in particular for time-resolved studies. They allow exploring the effect of peripheral nucleobases on processes triggered by UV radiation. The conclusions drawn from such studies regarding the role of terminal groups could be extrapolated, at a certain extent, to peripheral nucleobases directly attached to the guanine core, as part of loops. Moreover, they may be useful for the development of devices in the field of nanotechnology.

The objective of the present work is, then, to study the electron holes in (AG_4_A)_4_/K^+^. The first part deals with the spectral features of an electron hole in this system. As the absorption spectrum determined at pH 3 differs from that reported previously for tetramolecular G-Quadruplexes formed by the oligonucleotide TGGGGT [27], we present additional results obtained for the asymmetric structures (AG_4_)_4_/K^+^ and (G_4_A)_4_/K^+^, in which four adenines are located respectively, at the 5′ and the 3′ ends. Their electronic structure, as well as the electronic absorption spectra, are rationalized by Quantum Mechanical (QM)/Molecular Mechanics (MM) calculations based on the Density Functional Theory (DFT) and its time-dependent version (TD-DFT). In addition to the guanine core, four stacked adenines are included in the QM part; being the first time that all the peripheral groups at 5′ and 3′ ends are taken into account for the study of electron holes in tetramolecular G-Quadruplexes. For comparison, the dinucleotides 5′-AG-3′ and 5′-GA-3′ with stacked bases, are also computed. In the second part, we follow the evolution of the time-resolved absorption spectra obtained by photoionization of (AG_4_A)_4_/K^+^ at pH 7; we discuss the (G^+^)^●^ deprotonation dynamics and present evidence for the formation of a reaction product.

## 2. Results and Discussion

### 2.1. Spectral Properties

**Figure 2:**
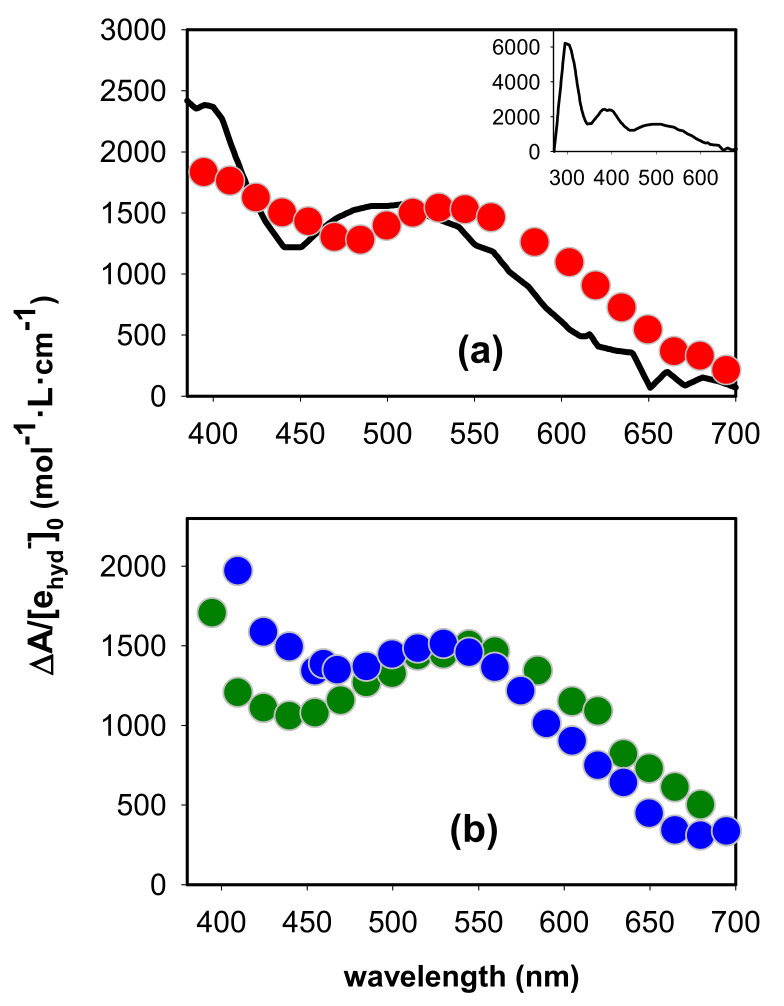
Experimental absorption spectra (circles) determined at 3 µs by photoionization of (**a**) the symmetric structure (AG_4_A)_4_/K^+^ (red) and (**b**) the two asymmetric analogues (G_4_A)_4_/K^+^ (green) and (AG_4_)_4_/K^+^ (blue) at pH 3; ∆A was divided by the initial concentration of hydrated ejected electrons. The black line in (**a**) corresponds to the absorption spectrum reported for the radical cation of dGMP [39], plotted with its molar absorption coefficient.

As the intensity of transient signals arising from guanine radicals is weak, spectra can be recorded only at times longer than 2 µs [29]. The spectrum obtained at 3 µs for (AG_4_A)_4_/K^+^ at pH 3 is compared with that reported for the (G^+^)^●^ in deoxyguanosine monophosphate (dGMP) in Figure 2a [33]. We note that this comparison is limited in the visible spectral domain because (G^+^)^●^ are very photoactive [38] in the UV. As a result, the probing light destroys the studied system due to the large number of accumulations required to obtain the G-Quadruplex spectrum. The monomer spectrum is represented with its molar absorption coefficient ε, while, in the case of G-Quadruplex, ΔA, determined over an optical path-length of 1 cm, was divided by the initial concentration of hydrated ejected electrons [e_hyd_^−^]_0,_ which is equal to that of generated electron holes. The methodology followed in order to determine [e_hyd_^−^]_0_ is described in reference [29].

We observe in Figure 2a that the intensity of the G-Quadruplex spectrum at 400 nm is significantly lower compared to that of the monomer. A similar trend was reported for the spectra of electron holes in monomolecular telomeric [26] and tetramolecular TGGGGT [27] G-Quadruplexes. However, for these two systems, the absorption band in the visible was found to practically overlap that of the monomeric (G^+^)^●^. This is not obviously the case for (AG_4_A)_4_/K^+^: although its band exhibits, within the experimental error (±5%), the same intensity as that of the monomeric (G^+^)^●^, its maximum is located at ~35 nm longer wavelengths (Figure 2a). Yet, the guanine cores of the tetramolecular structures formed by association of TGGGGT [40] and AGGGGA [36] strands have the same parallel topology; the cation spectra of these two systems are compared in Figure S1. We, therefore, conclude that it is the presence of adenines that alters the spectral properties of the electron hole.

The role of adenines on the spectrum of the electron hole was further checked by performing similar experiments on the asymmetric structures (AG_4_)_4_/K^+^ and (G_4_A)_4_/K^+^. The spectra of these two photoionized systems at pH 3 are clearly different (Figure 2b), that of (AG_4_)_4_/K^+^ being somewhat closer to the monomer spectrum. The spectrum of (AG_4_A)_4_/K^+^ appears, thus, to be an average of spectra of the two asymmetric G-Quadruplexes.

**Figure 3:**
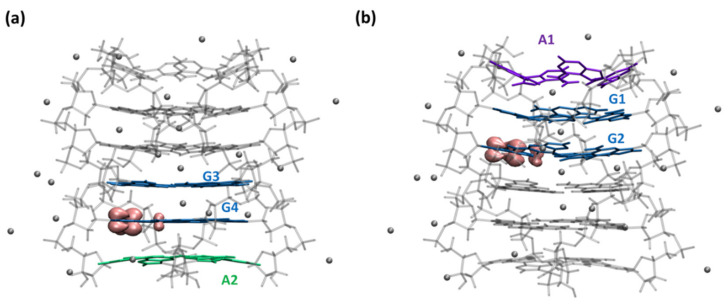
Spin densities for the optimized radical cation in the systems (**a**) (G_4_A)_4_/K^+^ and (**b**) (AG_4_)_4_/K^+^. MM guanines, backbone and ions are depicted in gray, QM guanines in blue, adenines in violet (at 5′) and green (at 3′).

In order to understand the origin of the adenine effect on the above presented spectra of electron holes, we performed theoretical calculations on (G_4_A)_4_/K^+^ and (AG_4_) _4_/K^+^ containing an electron hole. As a first step, we optimized their geometry. In this respect, we recall that two-dimensional NOESY NMR measurements on (AG_4_A)_4_/K^+^ did not detect any hydrogen bonding between adenines, meaning that no stable adenine tetrads are formed, but adenines could be intermittently stacked on the guanines [36]. Consequently, we considered the limiting case in which adenines are stacked on the guanine core since such an arrangement would maximize the electronic interactions between the two types of bases. Our computations showed that the electron holes are located at guanine sites for both the model dinucleotides (Figure S3) and the G-Quadruplexes (Figure 3). Although this is expected in view of the oxidation potentials of the bases, it is shown for the first time that this tendency is preserved even within G-Quadruplexes with stacked adenine nucleobase. Moreover, the cation is almost perfectly localized on a single guanine. This is surprising because theoretical studies reported hole delocalization for other parallel G-Quadruplexes [41], or even for simple parallel guanine stacks [42]. The structure of the optimized guanine cation within the systems studied here has the geometrical features reported for the monomeric species [42,43,44,45] and also found for previous G-Quadruplexes [26,27,41,46,47]. Despite the important shortening of the C2-NH2 bond in the guanine cation, the spin density has contributions from almost the entire ring of the nucleobase (Figure 3).

An important finding is that the electron hole in each asymmetric structure is not located on the same G-tetrad (Figure 3). In (G_4_A)_4_/K*^+^*, it is located within the G4 tetrad that is stacked with the 3′ adenines (A2), whereas in (AG_4_)_4_/K^+^, it is located in G2, corresponding to an inner tetrad, far from the 5′-adenines (A1). However, the relative energy difference between the two species is low, <1 kcal mol^−1^, suggesting that both are likely in the symmetric (AG_4_A)_4_/K^+^ structure with perfectly stacked adenines.

The existence of a radical cation in the guanine core of a G-Quadruplex, as part of an excited charge transfer (CT) state, is considered to be the primary step leading to low-energy photoionization [29,30,36]. We can rationalize our present findings in analogy with those previously reported regarding the nature and stability of CT states. It is nowadays well assessed, not only for G-Quadruplexes, but also for other DNA systems, that CT states are more stable when the electron moves along the 5′ → 3′ channel [36,48,49,50]. In the first place, this agrees with the “G-tetrad” in which the hole is located. If the system has a stacked 5′-GA-3′ sequence, the cation is localized close to the adenine connected with an initial stable 5’-G^+^ → A^−^-3′ CT state/minimum. However, adenines at the 5′ end would involve the formation of less stable 5’-A^+^ → G^−^-3′ (3′-G^+^ → A^−^-5) or 5′-G^+^ → G^−^-3′ CT states; thus, the cation tends to be localized at an inner tetrad. This matches our previous results about excited states in stacked AG/GA dinucleotides, which revealed that the coupling between the bright and CT states is larger in 5′-GA-3′ compared to 5′-AG-3′ [36]. The loss of stability when the non-guanine bases are positioned at the 5′ end is even more dramatic when adenine is replaced by thymine. For instance, non-CT minimum was located for the 5′-TG-3′ dinucleotide [36]. This could be related with the tendency towards some extent of delocalization between guanines for holes in tetramolecular G-Quadruplexes with thymine terminal groups [47].

**Figure 4:**
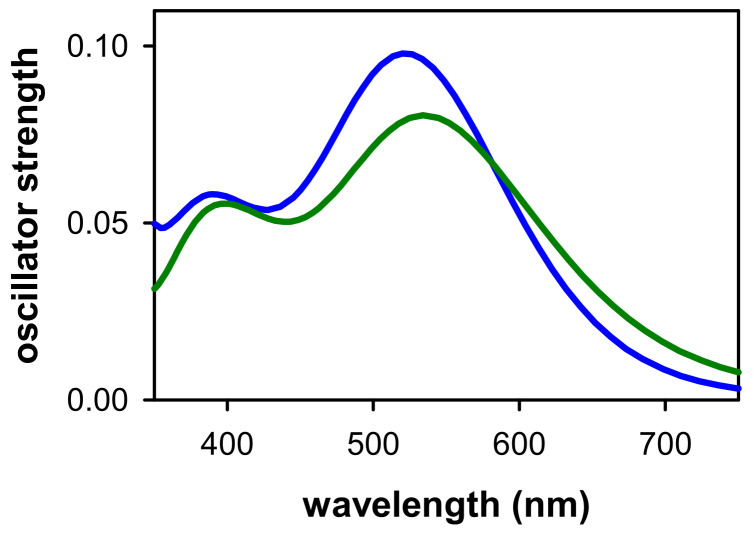
Absorption spectra computed for the optimized radical cation in (G_4_A)_4_/K^+^ (green) and (AG_4_)_4_/K^+^ (cyan) at the PCM/TD-DFT(M052X)/6-31G(d)//MM level of theory.

In a subsequent step, the absorption spectra of the two “cationic” G-Quadruplexes were modeled by computing the energies and intensities of the one hundred lowest-energy excited states obtained by TD-DFT (Figure 4). At wavelengths longer than 600 nm, the intensity found for the spectrum of the (G_4_A)_4_/K^+^ “cation” is higher compared to the (AG_4_)_4_/K^+^ analogue. A close inspection of the lowest-energy excited states reveals the potential background for this result. For the (AG_4_)_4_/K^+^ “cation”, the energies of these excited states are blue-shifted up to 0.2 eV and the global oscillator strength (f) is half the one summed up compared to (G_4_A)_4_/K^+^ “cation”. As discussed above, the most intense absorption of the (G_4_A)_4_/K^+^ “cation” in the red part of the spectrum stems from the directionality of the CT excited states: larger stability (i.e., lower energy) for the CT states in which the electron moves towards the 3′ end. The broad bands located in the 400–600 nm region follow similar trend in terms of energies, i.e., slightly red-shifted for the (G_4_A)_4_/K^+^ “cation”. However, the behavior of f is reversed compared to the red region, the (G_4_A)_4_/K^+^ “cation” spectrum being slightly less intense in the 500–550 nm region; although several excited states are present in this energy range, their computed f is at most 0.04. In contrast, the (AG_4_)_4_/K^+^ “cation” has two states peaking at these energies, one of them being particularly bright (f = 0.08).

Similar results were also obtained for the model dinucleotides (Figure S4): the lowest in energy broad band is red-shifted and less intense for 5′-GA-3′ compared to 5′-AG-3′ but these differences are smaller compared to those observed for G-Quadruplexes. This is related to the further stabilization (beyond directionality) of lower in energy CT excited states due to the presence of metal inner cations coordinated within G-Quadruplex systems [41,51,52,53].

### 2.2. Dynamical Properties

**Figure 5:**
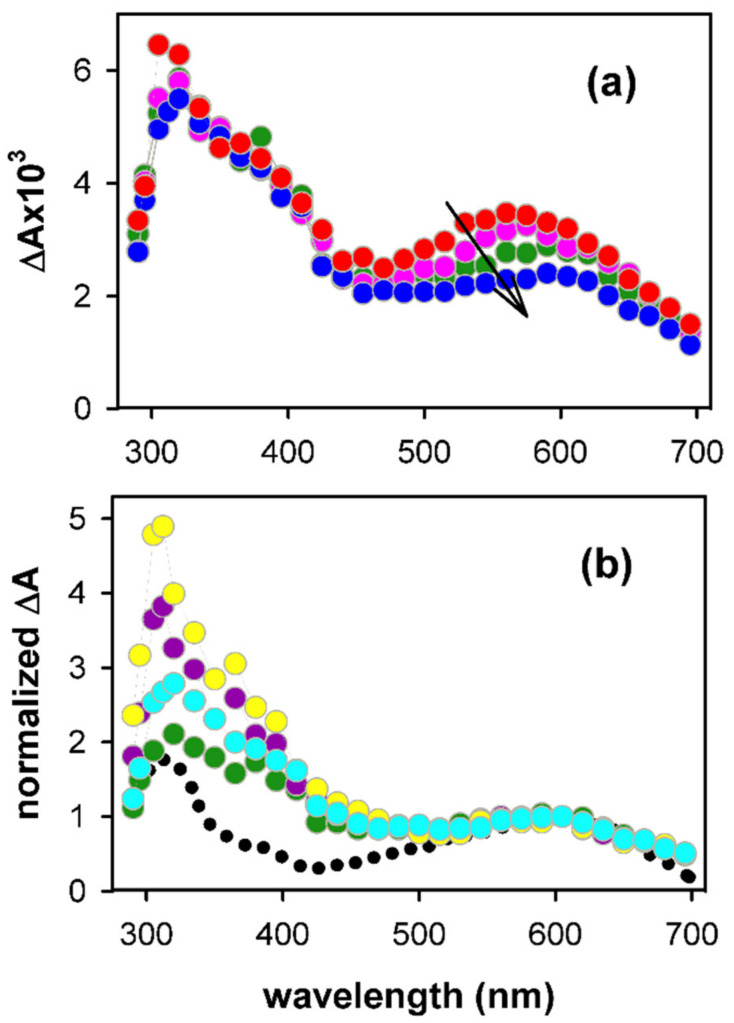
Transient absorption spectra determined for aerated solutions of (AG_4_A)_4_/K^+^ at pH 7 (circles) at 3 µs (red), 50 µs (pink), 0.2 ms (green), 0.5 ms (blue), 2 ms (cyan), 6 ms (dark red) and 10 ms (yellow). In (**a**), the experimentally measured ΔA is shown while in (**b**), ΔA at 605 nm has been normalized to 1; black dots correspond to the (G-H2)^●^ spectrum reported for monomeric guanosine [54].

The transient absorption spectra determined for (AG_4_A)_4_/K^+^ at pH 7 at selected times, ranging from 3 µs to 10 ms, are shown in Figure 5. The main evolution observed at early times is a shift of the band peaking in the visible toward longer wavelengths (Figure 5a). A similar trend was found for all the G-Quadruplexes studied so far [29,35]. It is attributed to the deprotonation of (G^+^)^●^, losing the amino proton at position 2, which is not engaged in hydrogen bonding [34] (see Figure 1a). As a result, the spectral feature of the deprotonated radical (G-H2)^●^, absorbing at around 600 nm (Figure 5b), becomes dominant.

**Figure 6:**
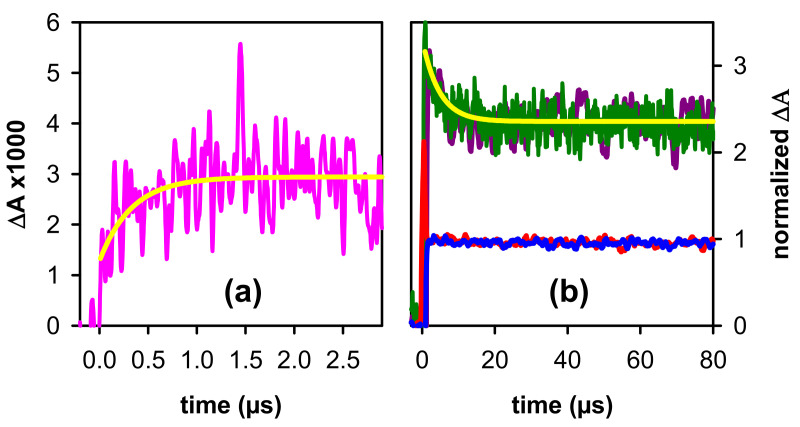
Transient absorption signals recorded for (AG_4_A)_4_/K^+^ at pH 7: (**a**) 605 nm (pink); N_2_O saturated solutions; (**b**) 290 nm (violet), 380 nm (blue), 530 nm (green) and 620 nm (dark red); aerated solutions. Yellow lines correspond to fits with mono-exponential functions: {A_1_ (1 − exp(t/τ_1_)) +A_0_ (rise)}and {A_1_ exp(−t/τ_2_) +A_0_} (decays); τ_1_ = 340 ± 40 ns and τ_2_ = 4.6 ± 0.4 µs.

As already described for other four-stranded systems [35], the (G^+^)^●^ decay in (AG_4_A)_4_/K^+^ is highly anisotropic, spanning from at least 30 ns to a few tens of µs. This is reflected on the rapid rise of the transient absorption signal observed on the sub-microsecond time-scale in the red part of the spectrum (Figure 6a), where the absorbance of (G-H2)^●^ radicals is higher than that of (G^+^)^●^. These experiments were performed by saturating the solution with N_2_O, an efficient scavenger of hydrated electrons [55], whose strong absorption blurs that of radicals [30]. However, as the sensitivity of the experimental setup is much lower at this time-domain, the transient signals are very noisy, despite the large number of accumulations. The rise in Figure 6a can be approximated by single exponential function with a time-constant of 340 ± 40 ns.

The (G^+^)^●^ population continues to decrease on the microsecond time-scale, as indicated by the signals recorded at wavelengths where its absorbance is preponderant: around its maximum (Figure 2a) and also below 300 nm, as expected in analogy with the spectrum of the monomeric cation (inset in Figure 2a). In this case, the signal has been approximated by a mono-exponential function (Figure 6b). We stress that the parameters derived from such fits do not correspond to reaction rate constants, as explained in detail in reference [35], but are simply used for a phenomenological description of the signals. In the red part of the spectrum, dominated by the absorbance of (G-H2)^●^, the transient signals appear to be flat. This could be explained by the superposition of a rise due to (G-H2)^●^ formation, and a decay due to the disappearance of (G^+^)^●^, also absorbing at this region. Similar quasi-flat signals are also observed around 350–400 nm.

**Figure 7:**
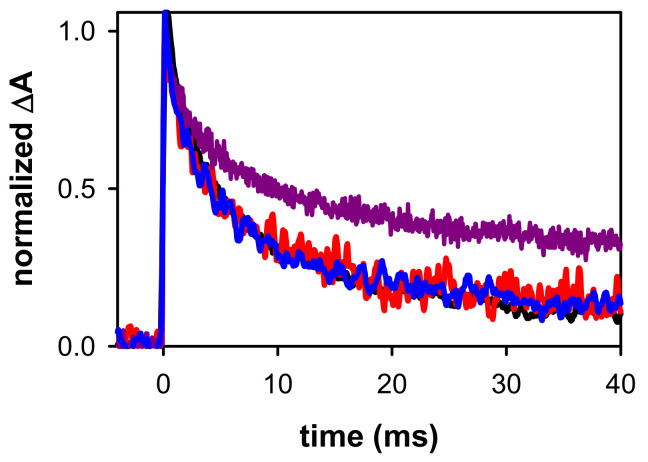
Normalized transient absorption decays determined for aerated solutions of (AG_4_A)_4_/K^+^ at 385 nm (violet), 500 nm (blue) and 685 nm (red). For comparison, the decay obtained for (TG_4_T)_4_/K^+^ at 605 nm [46] is shown in black.

In Figure 5b, we compare the evolution of the spectral shape recorded on the millisecond time-scale by normalizing the transient absorption around 600 nm. We observe that between 0.2 ms and 6 ms, the spectral shape in the 500–700 nm domain remains practically the same. In contrast, the relative intensity at shorter wavelengths, featuring a peak around 335 nm and a shoulder at ~380 nm, increases. We, therefore, conclude that, in addition to (G-H2)^●^, another species, noted as X, is formed. This is also attested by the transient absorption signals recorded on the millisecond time-scale: at all wavelengths above 500 nm, they practically overlap, while they follow a much slower decay pattern at shorter wavelengths (Figure 7). Moreover, we remark in Figure 7, that the decay of (G-H2)^●^ in (AG_4_A)_4_/K^+^ is similar to that observed for (TG_4_T)_4_/K^+^ [46], showing that their reaction rate does not depend on the nature of the terminal groups.

**Figure 8:**
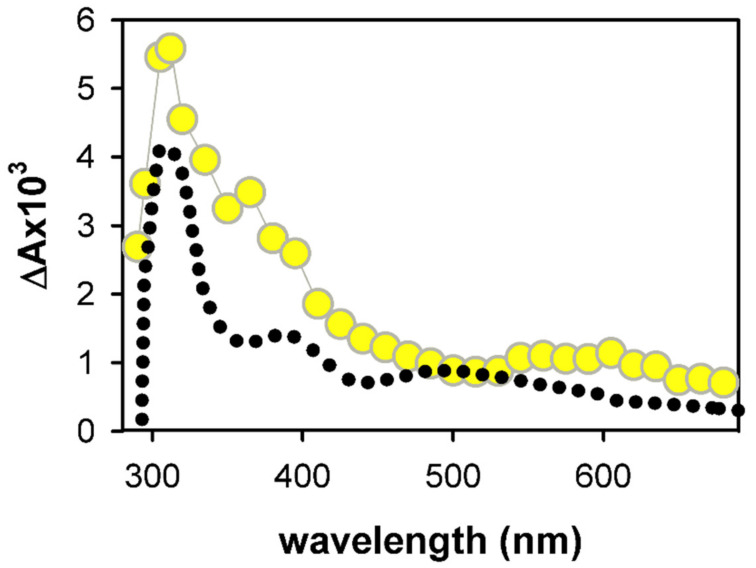
Comparison of the transient absorption spectrum determined for (AG_4_A)_4_/K^+^ at 10 ms (yellow circles) with that reported for monomeric (G-H1)^●^ (black dots) [39].

For several G-Quadruplexes studied previously, (G-H2)^●^ → (G-H1)^●^ tautomerization was observed on the millisecond time-scale [29]. This transformation is particularly clear in the case of monomolecular TEL21/Na^+^ and tetramolecular (TG_4_T)_4_/Na^+^ [26,27]. However, comparison of the spectrum obtained for (AG_4_A)_4_/K^+^ at 10 ms with that of monomeric (G-H1)^●^ in Figure 8, shows that tautomerization of deprotonated radicals does not constitute a major reaction pathway. At this point, we conclude that X is a reaction product or a reaction intermediate formed at earlier times and not correlated with (G-H2)^●^ radicals. The transient absorption experiments performed in the frame of this study dedicated to electron holes cannot unveil the nature of X. However, they provide some hints which may be useful for future works.

So far, the fingerprints of two types of UV-induced reactions in DNA have been detected in the 300–500 nm spectral region by nanosecond transient absorption spectroscopy. On the one hand, they are correlated with dimerization of nucleobases [31,32] occurring on a singlet excited state, i.e., at times much shorter than those explored by such experiments (see Figure 6). The initially formed photoproducts may also undergo further dark reactions on the millisecond time-scale. In all cases, the intensity of the transient signals is proportional to the concentration of the photons absorbed by the system [hν]. On the other hand, we may have reaction products or reaction intermediates resulting from photoionization. In this case, the intensity of their transient absorption is proportional to the concentration of electron holes generated within the system. Moreover, the latter is equal to the concentration of hydrated ejected electrons [e_hyd_^−^]_0_ which can be determined by independent experiments [29]. In the range of the excitation intensities used in the studies of low-energy DNA photoionization, e_hyd_^−^ results from both one- and two-photon processes. Thus, the behavior of the transient absorption observed upon changing the ratio [e_hyd_^−^]_0_/[ hν], achieved by varying the energy of the incident laser pulses, may help disentangling the two types of reaction.

**Figure 9:**
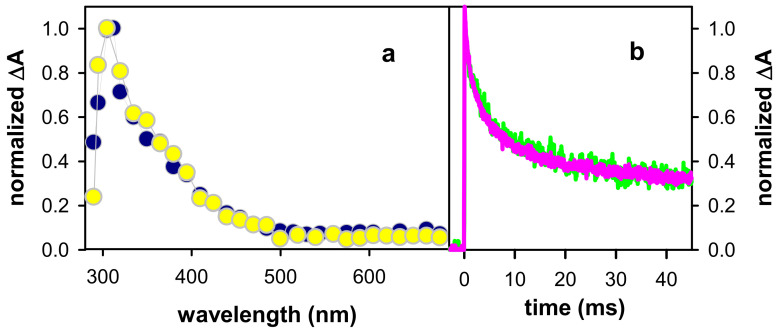
(**a**) Comparison of the transient absorption spectra determined for (AG_4_A)_4_/K^+^ (yellow circles) and TEL21/K^+^ (dark blue circles) at 30 ms; (**b**) Transient signals recorded for (AG_4_A)_4_/K^+^ at 365 nm with incident laser pulses: 3 mJ (green) and 6 mJ (pink).

The transient absorption spectrum recorded for (AG_4_A)_4_/K^+^ at 30 ms exhibits a striking similarity with that detected under the same conditions for TEL21/K^+^ [56] (Figure 9a). For the latter system, it was shown that it corresponds to a reaction intermediate decaying on hundreds of ms and stemming from photoionization because the pattern of transient absorption at 365 nm, where both X and (G-H2)^●^ absorb, is not altered upon varying [e_hyd_^−^]_0_/[ hν] by 50%. In the present study, the variation was limited to 20%, due to the lower contribution of the two-photon process to the (AG_4_A)_4_/K^+^ photoionization, as attested by the respective ionization curves [36,56]. Unfortunately, much larger variations of the laser pulse energy either lead to very noisy signals or provoke two-photons ionization of the aqueous solvent damaging the DNA [57]. Despite this limitation, we estimate that it would be possible to detect changes in the decay pattern if X had a different origin than (G-H2)^●^. However, this is not the case as shown in Figure 9b. Therefore, by analogy with the TEL21/K^+^ behavior, X could be a reaction intermediate resulting from electron holes.

Several types of DNA lesions are known to result from (G^+^)^●^ following reaction paths other than deprotonation; these include adducts with non-DNA species, as well as 8-oxo-7,8-dihydro-2′-deoxyguanosine (8-oxodG) [58]. Numerous publications discuss 8-oxodG formed in G-Quadruplexes through electron abstraction reactions with external oxidants (see, for example, references [59,60]). The formation of 8-oxodG was also explored for G-Quadruplexes that have been simply irradiated at 266 nm, in the absence of external oxidants. In the case of (TG_4_T)_4_/Na^+^ [27], which has the same topology as (AG_4_A)_4_/K^+^, no 8-oxodG was found using high performance liquid chromatography combined to mass spectrometry. In contrast, the same technique did detect 8-oxodG in photoionized TEL21/Na^+^ [26]. However, the time-resolved spectra recorded for latter system [26] do not exhibit the spectral features observed for (AG_4_A)_4_/K^+^ and TEL21/K^+^ (Figure 9), suggesting that a different reaction takes place in these systems.

## 3. Materials and Methods

### 3.1. Materials

Oligonucleotides were purchased from Eurogentec Europe. As the purity is an important requirement in radical studies, they were purified by reversed phase HPLC and tested by MALDI-TOF. The phosphate buffer was composed of an equimolar mixture of KH_2_PO_4_/K_2_HPO_4_ in concentrations 0.15 or 0.015 mol·L^−1^ each. Solutions were prepared using ultra-pure water, delivered by a Milli-Q Integral system. The pH of the buffer, measured by a HANNA Instr. Apparatus (pH 210), was adjusted to 7 by addition of a concentrated KOH solution. For measurements in pH 3, a concentrated solution of phosphoric acid (1 mol·L^−1^) was added in the solution. The purity of the buffer salts was 99.99%. For the formation of G-Quadruplexes, an appropriate mother solution was heated to 96 °C during 5 min, then slowly cooled to 4 °C, where it was incubated overnight. Prior to time-resolved experiments, the absorbance at 266 nm was adjusted to 2.0 ± 0.1 over 1 cm, heated to 50 °C, so that to destroy higher-order aggregates possibly formed at high concentration, and then cooled slowly to room temperature; the G-Quadruplex concentration was ~10^−5^ mol·L^−1^. G-Quadruplex formation was checked par circular dichroism (see SI in reference [36])

During the experiments, 2 mL of solution, contained in a 1 cm × 1 cm quartz cell, were mildly stirred while the temperature was maintained at 23 °C. To avoid re-excitation of damaged G-Quadruplexes, solutions were replaced frequently.

### 3.2. Transient Absorption Spectroscopy

The transient absorption setup used as excitation source was the fourth harmonic of a nanosecond Nd:YAG laser (Quanta Ray, Spectra-Physics, Santa Clara, CA, USA). The excited area at the surface of the sample was 0.6 × 1.0 cm^2^. The analyzing beam, orthogonal to the exciting beam, was provided by a 150 W Xe-arc lamp (OSRAM XBO, Munich, Germany); its optical path length through the sample was 1 cm while its thickness was limited to 0.1 cm in order to use the most homogeneous part of the light. The probed volume was located in the very first 0.1 cm part of the cell along the propagation of the exciting laser beam delimited by four dedicated slits. Then, the analyzing light was dispersed in a SPEX 270M monochromator (Horiba Jobin-Yvon, Longjumeau, France), detected by a Hamamatsu R928 photomultiplier (Hamamatsu Photonics France, Massy, France) and recorded by a Waverunner 6084 oscilloscope (Lecroy, Teledyn Lecroy, Courtaboeuf, France). For measurements on the sub-microsecond scale, the Xe-arc lamp was intensified via an electric discharge (Applied Photophysics, Leatherhead, UK). Transient absorption spectra were recorded using a wavelength-by-wavelength approach. The excitation rate was 0.2 Hz. The incident pulse energy at the surface of the sample was measured using a NIST traceable pyroelectric sensor (Nova2/PE25, Ophir Spiricon Europe GmbH, Darmstadt, Germany).

### 3.3. QM/MM Calculations

Our QM/MM ONIOM [61] models for the (G_4_A)_4_ and (AG_4_)_4_ systems are depicted in Figure 3. We included, in the QM part, the involved adenine tetrad (at 3′ or 5′ end) plus the two closest G-tetrads and inner ions. The rest of the system, i.e., the two remaining G tetrads, the backbones and the outer counterions are considered just at the MM level of theory. This allows for a balanced description of the asymmetric species with no dramatic effect on the selected geometry. The geometry of the “cationic” G-Quadruplexes was optimized at the QM/MM level, selecting density functional theory (DFT) and, in particular, the M052X [62,63] functional and the 6-31G(d) basis set for the QM region and amber force field parm96.dat [64] for the MM part. The entire system is considered to be embedded in water by using an implicit polarization method (Polarizable Continuum Model, PCM) [65,66]. For modeling the absorption spectra, we resort to the time-dependent version of DFT with the same settings above specified (TD-DFT) to compute the vertical absorption energies and oscillator strength. Then, these energies are uniformly shifted by −0.6 eV and convoluted with a Gaussian function with a hwhm of 0.3 eV. This computational approach has already been shown to satisfactorily reproduce the experimental absorption and circular dichroism spectra for other G-Quadruplex systems (see, for instance, reference [26,36,46,47,53]). All the calculations were performed using Gaussian16 program [67].

## 4. Main Conclusions

One important outcome derived from the computational part of our study is that the guanine site in which the electron hole is localized within the examined tetramolecular G-Quadruplexes is modulated by the stacking of adenines at the 3′ or 5′ ends. Although the energy difference determined for the two asymmetric models used in the computations is small, the situation may change in real systems under the effect of conformational motions and the intermittent stacking (and probably not symmetric) of the terminal groups. In a more general way, this is the first demonstration that peripheral bases may affect the position of the electron hole within the G-Quadruplex core. It would be interesting to pursue such investigations on monomolecular systems, more relevant for biological processes than the models examined here.

Peripheral adenine groups affect, in a different way, the absorption spectra of the electron hole, depending on their position in the 5′ or 3′ ends. Their knowledge could be useful for the characterization of excited CT by femtosecond spectroscopy [68], allowing to distinguish between G^+^ → G^−^ and G^+^ → A^−^ CT states. Such a disentanglement is important in order to better understand the mechanism responsible for low-energy photoionization of DNA [29,36]. Furthermore, it would also be interesting to explore by pulse radiolysis if similar effects are encountered for the corresponding “anionic” species formed by electron attachment [57,69].

As for the previously studied G-Quadruplexes, the decay of (G^+^)^●^ in (AG_4_A)_4_/K^+^ spans from at least 30 ns to tens of µs. It would not be surprising that such a highly anisotropic reaction rate arises, at least partly, from a variety of positions of electron holes within the G-Quadruplex core. (G^+^)^●^ deprotonation in (AG_4_A)_4_/K^+^ gives rise to (G-H2)^●^ deprotonated radicals, whose reaction products have not been identified so far. Our experimental results indicate that the peripheral groups are unlikely to be involved in the (G-H2)^●^ reactions because their decay pattern is the same for (AG_4_A)_4_/K^+^ and (TG_4_T)_4_/K^+^. Moreover, for both systems, (G-H2)^●^ → (G-H1)^●^ tautomerization does not constitute a noticeable reaction path.

Finally, our time-resolved experiments brought evidence for the formation of a reaction product, similar to that observed for the telomeric G-Quadruplex TEL21/K^+^, and probably stemming from (G^+^)^●^. Given that its spectral fingerprint decays, although more slowly than that of (G-H2)^●^, it should not correspond to a final lesion but rather to a reaction intermediate. However, the spectral and dynamical features presented here are not sufficient for its identification. We hope that our findings will motivate experts of analytical chemistry to focus on this problem.

## Data Availability

Not applicable.

## Supplementary Materials

Supplementary Materials can be found at https://www.mdpi.com/article/10.3390/ijms222413436/s1.
