# Supplementary material for: Electron Holes in G-Quadruplexes: The Role of Adenine Ending Groups
Source: Int J Mol Sci. 2021 Dec 14;22(24):13436. doi: 10.3390/ijms222413436 (PMC8704496; doi:10.3390/ijms222413436)
Supplement: Supplementary file 1 [file ijms-22-13436-s001.zip › ijms-1466024-supplementary.pdf]

# Electron holes in G-Quadruplexes: the role of adenine ending groups

**Evangelos Balanikas[1], Lara Martinez-Fernandez[2*], Gérard Baldacchino[1], and Dimitra Markovitsi[1*]**

[1] LIDYL, CEA, CNRS, Université Paris-Saclay, F-91191 Gif-sur-Yvette, France
[2] Departamento de Química, Modúlo 13, Facultad de Ciencias and IADCHEM (Institute for Advanced Research in Chemistry), Universidad Autónoma de Madrid, Campus de Excelencia UAM-CSIC, Cantoblanco, 28049 Madrid, Spain;

* Correspondence: lara.martinez@uam.es; dimitra.markovitsi@cea.fr

## Supporting Information

- Sequence effect on the G-Quadruplex "cation" spectra
- Scheme of  the exciting and probing beams
- Computational Results on Stacked Dinucleotides

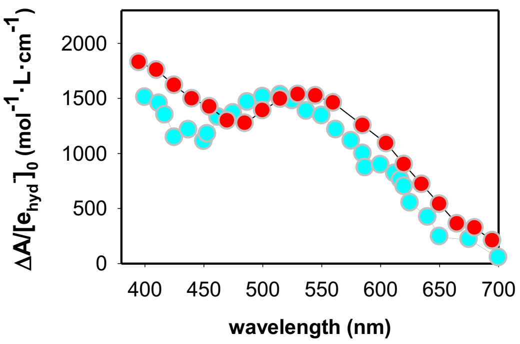


**Figure S1**. Sequence effect on the transient absorption spectra recorded for tetramolecular G-Quadruplexes at pH 3. Red : AGGGGA at 3 µs; cyan: TGGGGT at 2.5 µs (from reference [1] with ACS permission).

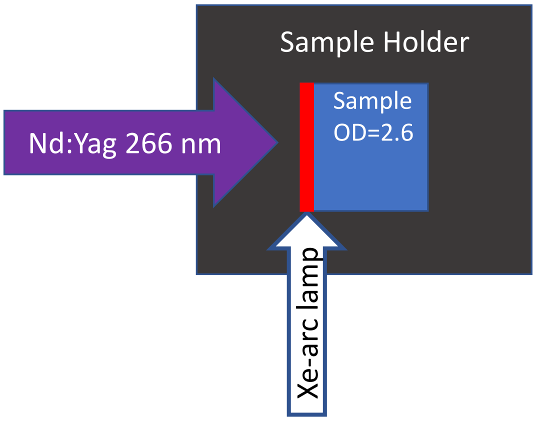


**Figure S2**. Schematic representation of the exciting and probing beams adopted in the nanosecond time-resolved experiments so that to determine weak transient absorption signals. In red: part of the solution which is effectively studied, the blue part serving as a reservoir.

## Computational results on stacked dinucleotides

Two dinucleotides (Figure S1) were optimized at full QM level using the same protocol as that used for G-Quadruplexes, i.e., PCM/M052X/6-31G(d). We considered the stacked guanine-adenine dimer at both 5′ and 3′ relative positions. First, we optimized the electron hole and found that, in both cases, it is localized in the guanine site (see Figure S1). Then, we computed the corresponding absorption spectra (Figure S2).

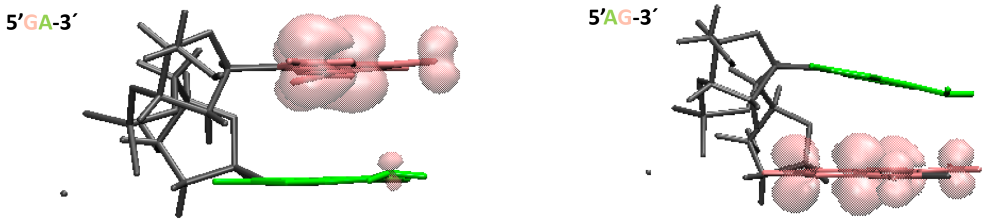


**Figure S3.** Spin densities for the optimized radical cation in the dinucleotides; pink: guanine; green: adenine; grey: backbone and counterion.

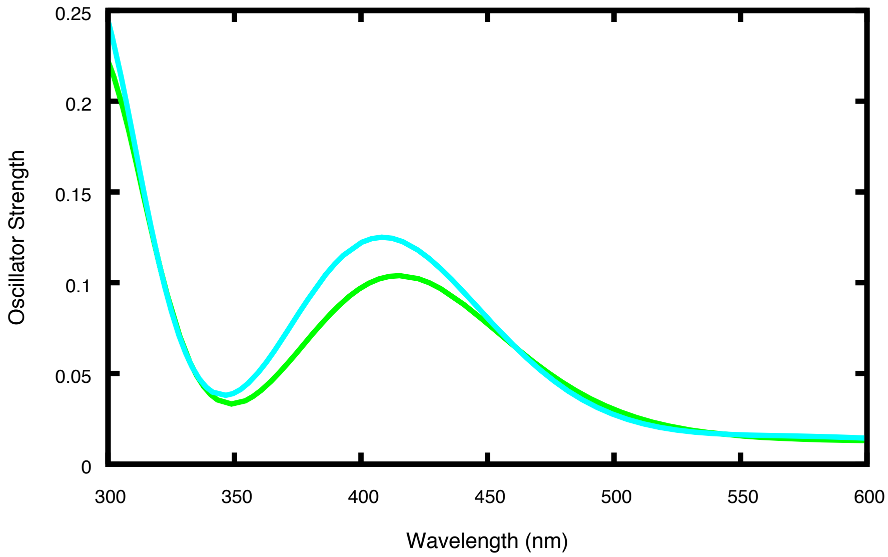


**Figure S4.** Absorption spectra computed for the optimized radical cation in GA (green) and AG (cyan) at the PCM/TD-DFT(M052X)/6-31G(d) level of theory.

1. Banyasz, A.; Balanikas, E.; Martinez-Fernandez, L.; Baldacchino, G.; Douki, T.; Improta, R.; Markovitsi, D. Radicals generated in tetramolecular guanine quadruplexes by photo-ionization: spectral and dynamical features. *J. Phys. Chem. B* **2019**, *123*, 4950-4957.
